# Determination of performance response of broilers to fermented tropical leaf meal supplementation using meta-analytical method

**DOI:** 10.1007/s11250-024-03944-w

**Published:** 2024-03-11

**Authors:** I. P. Ogbuewu, M. Mabelebele, C. A. Mbajiorgu

**Affiliations:** 1 Department of Animal Science and Technology, Federal University of Technology, P.M.B. 1526, Owerri, Imo State Nigeria; 2 Department of Agriculture and Animal Health, University of South Africa, Florida Science, Campus, Private Bag X6, Florida, 1710 South Africa

**Keywords:** Fermentation, Tropical leaf meals, Broilers, Performance, Product quality, Meta-analysis

## Abstract

Fermented tropical leaf meals (FTLM) are currently added to chicken feed to improve chicken productivity due to their reported nutritional and medicinal benefits. However, the effects of FTLM on broiler productivity and health are less clear. Thus, this meta-analysis was designed to assess the effects of FTLM on the performance outcomes of broilers Eleven controlled studies were retrieved and used to explore the impacts of dietary FTLM supplementation on growth performance [feed intake (FI), feed conversion ratio (FCR), average daily gain (ADG)], blood lipids, slaughter performance (abdominal fat, breast and thigh muscles weight), meat quality [pH, drip loss, shear force, lightness (L*), redness (a*), and yellowness (b*)], and intestinal histomorphology [villus height (VH), crypt depth (CD) and VH/CD values] of broilers. Subgroup and meta-regression analyses of the effects of moderators (i.e., leaf meal type, supplementation level, broiler strains, rearing phase, and fermentation microbes) on the growth performance of broilers were also assessed. Results show that dietary FTLM supplementation increased FI [standardised mean difference (SMD) = 0.11; 95% confidence interval (CI): 0.02, 0.20; *P* < 0.0001], improved ADG (SMD = 0.33; 95% CI: 0.23, 0.43; *P* < 0.0001) and FCR (SMD =  − 0.21; 95% CI: − 0.30, − 0.11; *P* < 0.0001) in broilers. In addition, FTLM enhanced slaughter performance, meat quality, and intestinal histomorphology of broilers. Broilers fed 0—5 g/kg feed FTLM had better FI, FCR, and ADG than the controls taking significant heterogeneity into account. Meta-regression revealed that analysed moderators influenced growth performance results and accounted for some of the sources of heterogeneity. It can be concluded that up to 5 g/kg of FTLM can be added to broiler feed to improve growth performance, intestinal histomorphology, slaughter performance, and meat quality without adverse effects on dressing percentage and blood lipid profiles.

## Introduction

Due to the escalating cost of conventional feedstuffs, leaf meals from tropical plants are now added to poultry feed to reduce production costs and improve productivity and product quality (Manyelo et al. 2022; Niu et al. 2023). Tropical climate is endowed with a diversity of plants that can yield large leaf biomass throughout the year (Okoli et al. 2019). Leaf meals of tropical plants are high in essential nutrients (vitamins, crude proteins, and minerals), polysaccharides, and bioactive components such as polyphenols and flavonoids (Mat et al. 2020; Shi et al. 2021; Sugiharto 2021). Researchers have demonstrated that supplementation of high levels of tropical leaf meals (TLM) in the chicken diet results in poor performance and product quality (Modisaojang-Mojanaja et al. 2019; Ogbuewu and Mbajiorgu 2019; Manyelo et al. 2022). The reasons for the very poor performance of chickens fed higher levels of TLMs are not clear but may be ascribed to their high crude fibre content, imbalance amino acid profile, poor digestibility, and the presence of anti-physiological agents. It is therefore vital to explore ways of adding more values to TLMs for optimal results as is the case with other feed resources high in crude fibre (Chukwukaelo et al. 2018; Ahiwe et al. 2022).

Chemically, the bioactive components in tropical leaves are polyphenols, flavonoids, organic acids, and terpenoids (Cao et al. 2012; Niu et al. 2017), which contribute to their antimicrobial and antioxidative activities. Research has shown that flavonoids isolated from tropical plants have positive effects on lipid metabolism which may be related to their antioxidant properties (Feng et al. 2011; Cao et al. 2012; Arora and Itankar 2018). Studies have confirmed that TLMs have the following pharmacological activities: anti-inflammatory, antiplatelet, antiviral, anticancer, neuro-protection, and cardio-protection effects (Liu et al. 2013; Niu et al. 2017; Amodeo et al. 2019). The feeding value of TLMs is influenced by their low to moderate levels of anti-nutritional factors, such as trypsin inhibitors, pectin, phytate, saponins, tannins, and several others (Pachauri et al. 2017).

The use of fermentation as a method for increasing the digestibility of novel feed resources has been stated (Sugiharto and Ranjitkar 2019; Shi et al. 2021). Fermentation has been found to improve nutritional and functional values of TLMs as well as enhance performance parameters and product quality of broilers (Sugiharto and Ranjitkar 2019; Shi et al. 2021). Fermented TLM has also been reported to improve productivity of animals other than broilers (Zhou et al. 2015; Lee et al. 2019). However, there are variable results on the impacts of dietary fermented TLM on broiler productivity and meat quality (Cao et al. 2012; Mandey et al. 2015; Santoso et al. 2015, 2018; Kim et al. 2017; Manihuruk et al. 2018; Niu et al. 2019), and this discrepancy could be attributed to differences in broiler genetics, microorganisms implicated in the fermentation process, substrate used, composition of TLM, quantity of TLM incorporated into the diet and several other factors known to affect chicken performance (Ditengou et al. 2023; Ogbuewu and Mbajiorgu 2023). Thus, it is pertinent to systematically summarise the findings of published studies that investigated the effects of FTLM on broiler chicken performance and product quality to enhance the use of these large volumes of information in evidence-based decision-making process. This study, therefore aimed to evaluate the meta-analytic effect of FTLM supplementation on growth performance, blood lipids, intestinal histomorphology, slaughter performance, and meat quality of broilers.

## Materials and methods

### Search strategy and study selection process

**Fig. 1:**
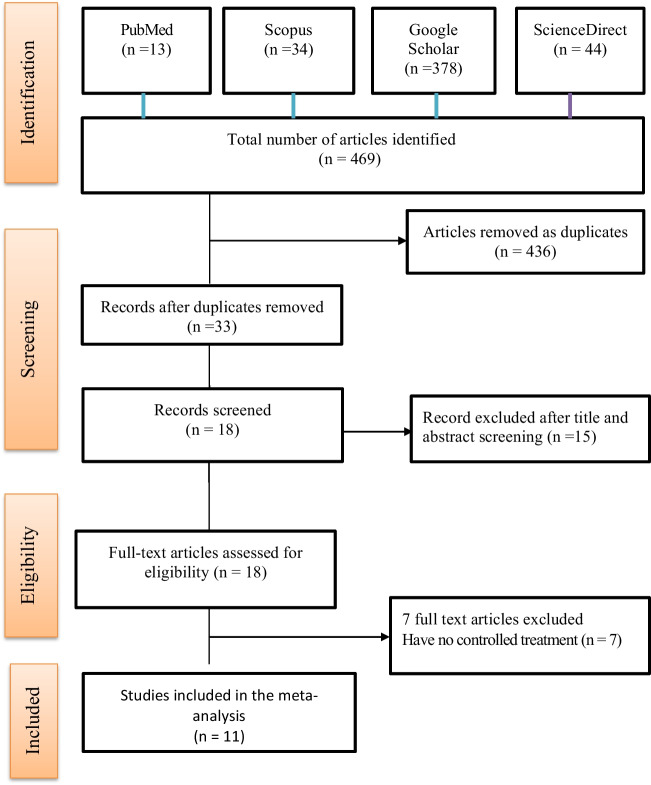
Article selection flow chart

PubMed, Scopus Google Scholar, and ScienceDirect databases were searched for articles on the topic using combinations of search terms and queries following the guideline for reporting systematic reviews and meta-analyses (Moher et al. 2009). Four hundred and sixty-nine (469) articles were identified via searches performed on the four databases of which eleven met the selection criteria (Fig. 1).

### Data extraction and synthesis

Information on author name, publication year, study country, and moderators: broiler strains (Arbore Acres yellow feathered and Lohmann), leaf meal type (*Ginkgo biloba*, *Morus alba*, *Sauropus androgynus* and *Chenopodium album*), fermentation microbes (*Lactobacillus spp*, *Bacillus subtilis*, *Aspergillus niger*, *Candida utilis*, *B. licheniform*. *Lactobacillus plantarum, Trichoderma harzianum, Neurospora crassa* and *Saccharomyces cerevisiae*), supplementation level (0 – 14 g/kg feed), and rearing phase (starter, finisher and overall) was extracted from eligible studies (Table 1) and entered into a Microsoft Excel sheet. Supplementation level was further subdivided into 0—5, 6—10, and > 11—14 g/kg feed. The rearing phase comprised the starter phase (0—21 days), finisher phase (22–56 days), and overall phase (0–56 days). Extracted outcome data were FI, ADG, FCR, intestinal histomorphology (VH, CD and VH/CD ratio), blood lipids [cholesterol, triglycerides, high-density lipoprotein (HDL), and low-density lipoprotein (LDL)], slaughter performance (abdominal fat, breast, thigh, and dressing percentage), and meat quality parameters (pH, drip loss, shear force, L*, a*, and b*). Data were also extracted on the means and standard deviation (SD) or standard error (SE) for the control and experimental groups for each measured outcome. The extracted data was converted to a comma-separated value (CSV) file format which is the format accepted by OpenMEE software. Where a trial stated SE instead of SD, the SE was transformed to SD using the formula of Higgins and Deeks (2011): SD = SE × √n, where n is the number of broilers included in each treatment group.

**Table 1:** Characteristics of studies used for the meta-analysis

Authors	Country	Explanatory moderator variables					Outcomes
		FM	Broiler strain	RP (days)	Leaf type	Inclusion (g/kg)	
Cao et al. (2012)	China	*A. niger*	Arbor Acres	1–21; 22–42; 1–42	*G. biloba*	0, 2 – 10	GP, AF, CC,BL, MQ
Zhang et al. (2012)	China	*A. niger*	Arbor Acres	1–21; 22–42; 1–42	*G. biloba*	0, 2 – 10	GP, SIH
Syahruddin et al. (2013)	Indonesia	*T. harzianum*	Lohmann	1–56	*S. androgynus*	0, 2—14	GP
Santoso et al. (2015)	Indonesia	+	-	15–35	*S. androgynus*	0. 2.5 -5.0	BL
Zhang et al. (2015)	China	^*^	Arbor Acres	1–21; 22–42; 1–42	*G. biloba*	0, 5	GP, SIH
Yu et al. (2015)	China	^**^	Arbor Acres	0–21; 22–42; 0–42	*G. biloba*	0, 3.5	GP, SIH
Niu et al. (2017)	China	^***^	Arbor Acres	1–21; 22–42; 1–42	*G. biloba*	0, 1.5—5.5	GP, AF, CC, MQ
Niu et al. (2019)	China	^***^	Arbor Acres	1–21; 22–42; 1–42	*G. biloba*	0, 1.5 – 5.5	GP
Zhang et al. (2020)	China	*A. niger*	Arbor Acres	1–21; 22–42; 1–42	*G. biloba*	0, 1—6	GP
Ding et al. (2021)	China	+ +	YFB	1–28, 29–56; 1–56	*M. alba*	0, 3—9	GP, SIH, AF, CC, MQ
Xie et al. (2021)	China	+ + +	Arbor Acres	1–21; 22–42; 1–42	*C. album*	0, 2 – 8	GP, CC, AF, MQ

^*^Candida utilis, *Aspergillus niger* and their mixture

^**^Bacillus subtilis var. natto or *Bacillus licheniformis*

^***^ mixture of *Candida utilis* and *Aspergillus niger*

+ *Neurospora crassa*, *Lactobacillus spp*. or *Saccharomyces cerevisiae*

+  + 1:2:1 mixture of Lactobacillus, Saccharomycetes and *Bacillus subtilis*

+  +  + mixture of *B. subtilis*, *L. plantarum* and *S. cerevisiae*

*FM* Fermentation microbes

*RP* Rearing phase

*AF* Abdominal fat

*YFB* Yellow feathered broilers

*GP* Growth performance (i.e., feed intake, feed conversion ratio and average daily gain)

*CC* Carcass characteristics (dressing percentage, breast muscle weight and thigh muscle weight)

*BL* Blood lipids (i.e., cholesterol, triglycerides, high—density lipoprotein cholesterol, and low- density lipoprotein cholesterol)

*SIH* Small intestine histomorphology (villi height, crypt depth and villi height/crypt depth ratio of the duodenum, jejunum and ileum)

*MQ* Meat quality (pH, drip loss, shear force, L*, a* and b* values of the breast and thigh muscles)

### Data analysis

Statistical analyses were executed in OpenMEE software designed and built by Wallace et al. (2016) at *P* < 0.05%. The execution flow chart of OpenMEE software has been described by Ogbuewu and Mbajiorgu (2020). Hedges’d commonly known as SMD was used to determine the pooled effect of FTLM on measured outcomes in broilers, and results were expressed at 95% confidence interval (CI). A random-effects model was used based on the assumption that the data being analysed are drawn from a hierarchy of different populations (Ogbuewu et al. 2022). Cochran’s Q-statistic and I^2^-test were used to assess and quantify statistical heterogeneity (Higgins et al. 2003). The meta-regression test was performed to determine the percentage of heterogeneity explained by analysed moderators. Publication bias was carried out to ascertain the validity of the results of the meta-analysis using Rosenberg’s fail-safe number (Rosenberg 2005). Pooled results were considered robust despite the evidence of publication bias if Nfs > 5 × n + 10, where, n = dataset (Jennions et al. 2013).

## Results

### Growth performance

**Fig. 2:**
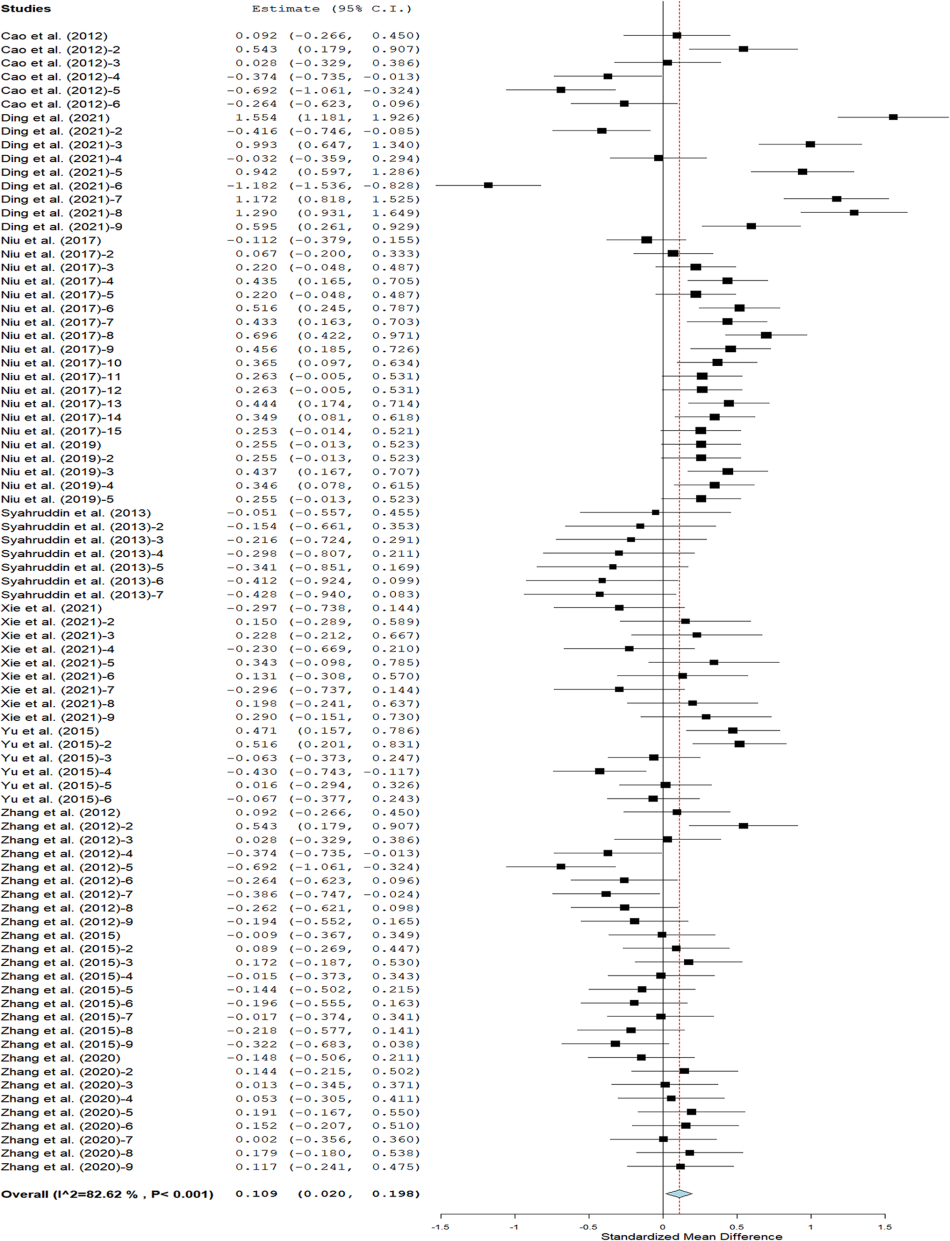
Feed intake of broilers on dietary FTLM intervention

The pooled mean FI as presented in Fig. 2 showed that broilers fed FTLM had higher values than the control. The subgroup analyses of the effect of moderators on FI are shown in Table 2. Arbor Acres strain offered FTLM supplementation had significantly higher FI than the Lohmann strain, but had similar FI with the Yellow feathered strain. Broilers fed FTLM at 0—5 g/kg feed recorded higher FI than the control. Leaf meal type had effect on FI, with broilers on fermented *G. biloba* leaf meal having significantly higher FI than those on fermented *S. androgynus* leaf meal. Starter broilers fed FTLM recorded higher FI than the control. In comparison to the controls, broilers fed TLM fermented with *T. harzianum* had significantly reduced FI while those fed diet containing fermented blends of *A. niger* and *C. utilis* had statistically increased FI. In contrast, broilers fed TLM fermented with *A. niger, B. subtilis*, *B. licheniform*, *C. utilis*, blends of Lactobacillus, Saccharomycetes, and *B. subtilis*, mixtures of *B. subtilis*, *L. plantarum* and *S. cerevisiae* had comparable FI with the control.

**Table 2:** Feed intake of broilers on dietary FTLM intervention

Outcomes	Random-effects model						Heterogeneity	
	Subgroup	Dataset	SMD	95% CI		*P* value	I^2^	*P* value
Broiler strain	Arbor acres	68	0.09	0.02	0.16	0.013	69	< 0.001
	YF	9	0.55	− 0.04	1.13	0.067	96	< 0.001
	Lohmann	7	− 0.27	− 0.46	-0.08	0.006	0	0.944
SL (g/kg)	0—5	54	0.13	0.06	0.21	< 0.001	65	< 0.001
	6—10	14	0.02	− 0.17	0.19	0.873	74	< 0.001
	11—14	16	0.20	− 0.21	0.61	0.343	94	< 0.001
LMT	*G. biloba*	59	0.09	0.02	0.17	0.017	71	< 0.001
	*M. alba*	9	0.55	− 0.04	1.13	0.067	96	< 0.001
	*S. androgynus*	7	− 0.27	− 0.46	− 0.08	0.006	0	0.944
	*C. album*	9	0.06	− 0.11	0.23	0.503	24	0.230
RP	Starter	25	0.23	0.07	0.38	0.003	80	< 0.001
	Finisher	25	− 0.02	− 0.21	0.17	0.828	87	< 0.001
	Overall	34	0.12	− 0.01	0.26	0.061	78	< 0.001
FM Microbe	*A. niger*	27	− 0.06	− 0.18	0.05	0.264	62	< 0.001
	*	9	0.55	− 0.04	1.13	0.067	96	< 0.001
	**	23	0.28	0.19	0.36	< 0.001	55	0.001
	*T. harzianum*	7	− 0.27	− 0.46	− 0.08	0.006	0	0.944
	***	9	0.06	− 0.11	0.23	0.503	24	0.230
	*B. subtilis*	3	0.14	− 0.18	0.47	0.396	69	0.038
	*B. licheniform*	3	0.01	− 0.53	0.54	0.983	89	< 0.001
	*C. utilis*	3	− 0.01	− 0.22	0.19	0.898	0	1.00

^*^: Lactobacillus + Saccharomycetes + *B. subtilis;* **: *A. niger* + *C. utilis*; ***: *B. subtilis* + *L. plantarum* + *S. cerevisiae*; *RP* Rearing phase; *FTLM* Fermented tropical leaf meal; *SL* Supplementation level; *FM* Fermentation microbes; *LMT* Leaf meal type; *SMD* Standardised mean difference; *YF* Yellow feathered; *HDL* High-density lipoprotein; *LDL* Low-density lipoprotein; *CI* Confidence interval. *I*^*2*^ Inconsistency index; Q *χ*^*2*^ statistic; τ^2^ Heterogeneity variance of the true effect sizes; *p* Probability

**Fig. 3:**
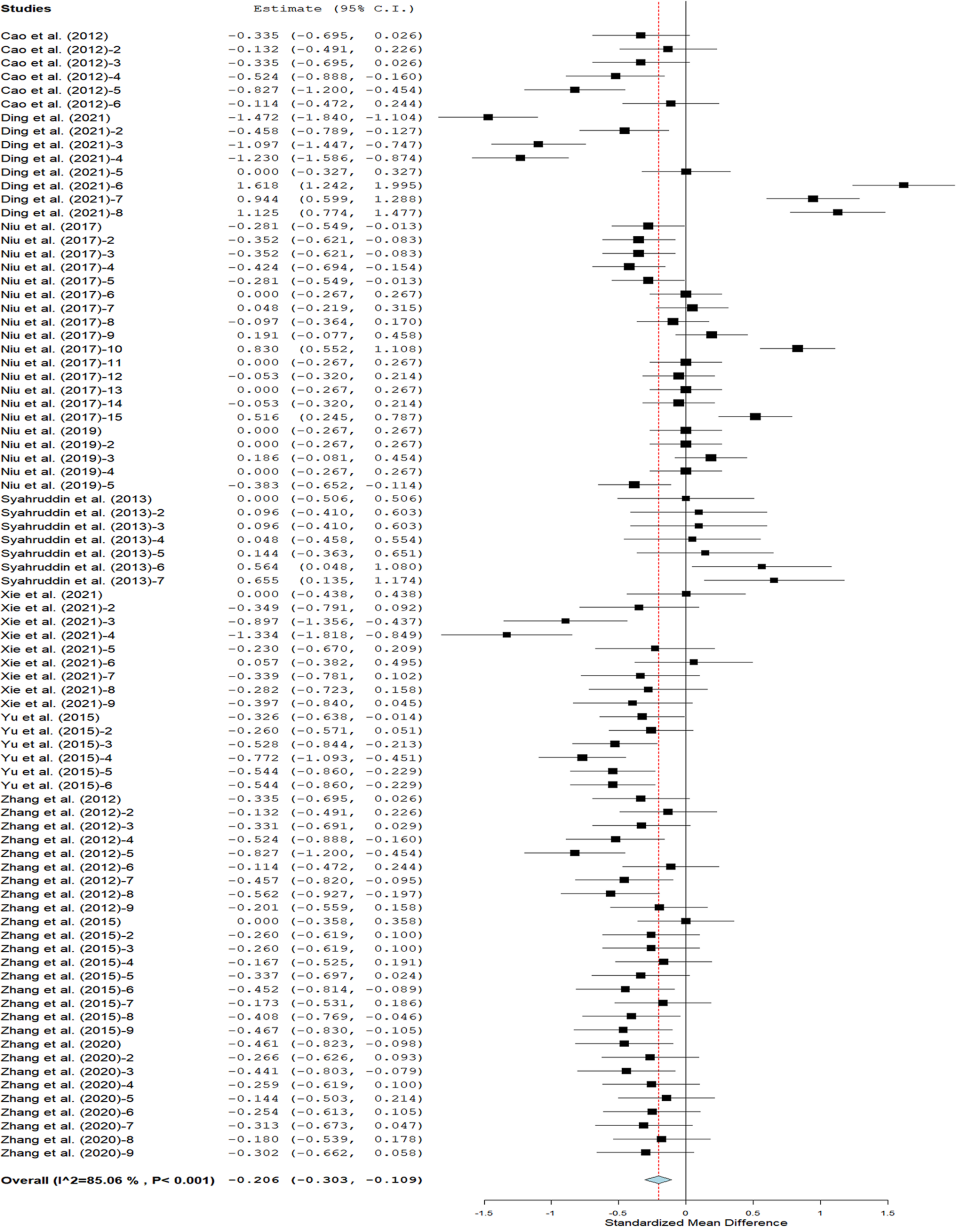
Feed conversion ratio of broilers on dietary FTLM supplementation

As displayed in Fig. 3, a significant difference occurred in FCR (SMD =  − 0.21; 95% CI: − 0.30, − 0.11; *P* < 0.001) between broilers on treatment and control diets. Results of subgroup analyses of the impact of moderators on FCR are presented in Table 3. There was no effect of broiler strains on FCR between broilers in control and treatment groups; however, superior FCR was reported in Arbor Acres and Lohmann strains when compared with control. Broilers offered FTLM at 0 – 5 g/kg feed had better FCR than the control. Broilers fed fermented *G. biloba* leaf meal had better FCR (SMD =  − 0.23; 95% CI: − 0.31, − 0.16) than those that received *M. alba* leaf meal (SMD =  − 0.07; 95% CI: − 0.87, − 0.73) and *S. androgynus* leaf meal (SMD = 0.22; 95% CI: 0.03, 0.42). Also, broilers fed *S. androgynus* leaf meal had poor FCR (SMD = 0.22; 95% CI: 0.03, 0.42) compared to those offered *M. alba* leaf meal (SMD =  − 0.07; 95% CI: − 0.87, − 0.73). Starter and finisher broilers fed FTLM recorded lower FCR than the control. Broilers fed TLM fermented with *A. niger* had better FCR than broilers offered TLM fermented with *T. harzianum*. Similarly, broilers fed TLM fermented with *B. subtilis*, *B. licheniform*, *T. harzianum* and blends of *B. subtilis*, *L. plantarum,* and *S. cerevisiae* had better FCR than the control.

**Table 3:** Feed conversion ratio of broilers on dietary FTLM supplementation

Outcomes	Random-effects model						Heterogeneity	
	Subgroup	Dataset	SMD	95% CI		*P* value	I^2^	*P* value
Broiler strain	Arbor acres	68	− 0.22	− 0.33	− 0.18	< 0.001	71	< 0.001
	YF	8	− 0.07	− 0.87	0.72	0.860	98	< 0.001
	Lohmann	7	0.22	0.03	0.42	0.024	2	0.412
SL (g/kg)	0—5	54	− 0.25	− 0.31	− 0.18	< 0.001	53	0.001
	6—10	14	− 0.24	− 0.51	0.034	0.091	89	< 0.001
	11—14	15	0.07	− 0.42	0.55	0.792	95	< 0.001
LMT	*G. biloba*	59	− 0.23	− 0.31	− 0.16	< 0.001	71	< 0.001
	*M. alba*	8	− 0.07	− 0.87	− 0.73	0.860	98	< 0.001
	*S. androgynus*	7	0.22	0.03	0.42	0.024	2	0.412
	*C. album*	9	− 0.41	− 0.69	− 0.14	0.003	70	0.001
RP	Starter	22	− 0.31	− 0.38	− 0.24	< 0.001	0	0.789
	Finisher	27	− 0.32	− 0.54	− 0.09	0.006	92	< 0.001
	Overall	34	− 0.05	− 0.18	0.09	0.517	81	< 0.001
FM	*A. niger*	27	− 0.34	− 0.41	− 0.27	< 0.001	3	0.424
	*	8	− 0.07	− 0.87	0.72	0.860	98	< 0.001
	**	23	− 0.07	− 0.19	0.06	0.288	79	< 0.001
	*T. harzianum*	7	0.22	0.03	0.42	0.024	2	0.412
	***	9	− 0.41	− 0.69	− 0.14	0.003	70	0.001
	*B. subtilis*	3	− 0.47	− 0.65	− 0.28	< 0.001	0	0.561
	*B. licheniform*	3	− 0.52	− 0.81	− 0.23	< 0.001	60	0.080
	*C. utilis*	3	− 0.11	− 0.32	0.09	0.284	0	0.749

^*^: Lactobacillus + Saccharomycetes + *B. subtilis;* **: *A. niger* + *C. utilis*; ***: *B. subtilis* + *L. plantarum* + *S. cerevisiae*; *RP* Rearing phase; *FTLM* Fermented tropical leaf meal; *SL* Supplementation level; *FM* Fermentation microbes; *LMT* Leaf meal type; *SMD* Standardised mean difference; *YF* Yellow feathered; *HDL* High-density lipoprotein; *LDL* Low-density lipoprotein; *CI* Confidence interval. *I*^*2*^ Inconsistency index; Q *χ*^*2*^ statistic; τ^2^ Heterogeneity variance of the true effect sizes; *p* Probability

**Fig. 4:**
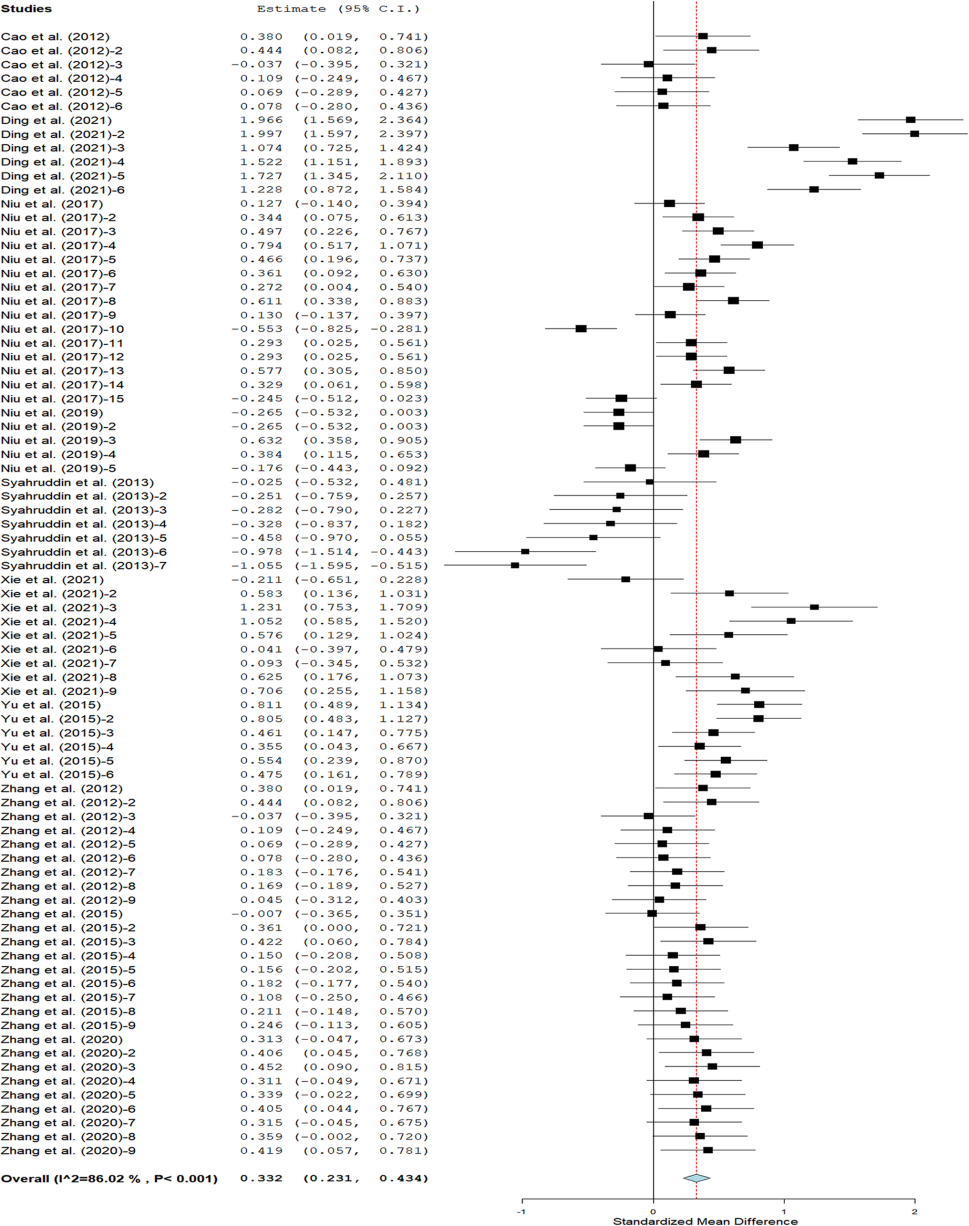
Average daily gain of broilers on dietary FTLM intervention

The ADG as displayed in Fig. 4 suggests that broilers fed FTLM gained more weight than the control broilers. The restricted subgroup analyses of the effect of moderators on ADG as shown in Table 4 indicate that Arbor Acres and Yellow feathered broilers fed FTLM at 0 – 5 g/kg feed had heavier ADG than the Lohmann broiler strain. Similarly, broilers that received fermented leaf meals of *G. biloba*, *M. alba,* and *C. album* gained more weight than those that received fermented *S. androgynus* leaf meal. Broilers fed TLM fermented with a mixture of *A. niger* and *C. utilis* had superior ADG than those fed diet fermented with *A. niger, T. harzianum, B. subtilis*, *B. licheniform, C. utilis,* and blends of *B. subtilis*, *L. plantarum,* and *S. cerevisiae*.

**Table 4:** Average daily gain of broilers on dietary FTLM supplementation

Outcomes	Random-effects model						Heterogeneity	
	Subgroup	Dataset	SMD	95% CI		*P* value	I^2^	*P* value
Broiler strain	Arbor acres	68	0.29	0.22	0.37	< 0.001	70	< 0.001
	YF	6	1.58	1.27	1.88	< 0.001	75	0.001
	Lohmann	7	− 0.47	− 0.76	− 0.19	< 0.001	52	0.052
SL (g/kg)	0—5	54	0.33	0.26	0.40	< 0.001	61	< 0.001
	6—10	14	0.20	− 0.03	0.42	0.083	83	< 0.001
	11—14	13	0.49	− 0.11	1.08	0.107	96	< 0.001
LMT	*G. biloba*	59	0.27	0.19	0.34	< 0.001	68	< 0.001
	*M. alba*	6	1.58	1.27	1.88	< 0.001	75	0.001
	*S. androgynus*	7	− 0.47	− 0.76	-0.19	< 0.001	52	0.052
	*C. album*	9	0.52	0.21	0.82	< 0.001	76	< 0.001
RP	Starter	22	0.41	0.28	0.53	< 0.001	86	< 0.001
	Finisher	26	0.45	0.24	0.67	< 0.001	90	< 0.001
	Overall	33	0.19	0.03	0.35	0.023	86	< 0.001
FM	*A. niger*	27	0.24	0.171	0.31	< 0.001	0	0.806
	*	6	1.58	1.27	1.88	< 0.001	75	0.001
	**	23	0.24	0.10	0.38	0.001	83	< 0.001
	*T. harzianum*	7	− 0.47	− 0.76	− 0.19	< 0.001	52	0.052
	***	9	0.52	0.21	0.82	< 0.001	76	< 0.001
	*B. subtilis*	3	0.61	0.40	0.81	< 0.001	20	0.289
	*B. licheniform*	3	0.54	0.28	0.80	< 0.001	51	0.128
	*C. utilis*	3	0.08	− 0.12	0.29	0.429	0	0.821

^* ^Lactobacillus + Saccharomycetes + *B. subtilis;* ** *A. niger* + *C. utilis*; *** *B. subtilis* + *L. plantarum* + *S. cerevisiae*; *RP* Rearing phase; *FTLM *Fermented tropical leaf meal; *SL* Supplementation level; *FM* Fermentation microbes; *LMT* Leaf meal type; *SMD* Standardised mean difference; *YF* Yellow feathered; *HDL* High-density lipoprotein; *LDL* Low-density lipoprotein; *CI* Confidence interval. *I*^*2*^ Inconsistency index; Q *χ*^*2*^ Statistic; τ^2^ Heterogeneity variance of the true effect sizes; *p* Probability

### Blood lipid profiles and intestinal histology

Data on blood lipids of broilers fed FTLM are shown in Table 5. Fermented TLM had a reduction effect on blood cholesterol content, although the effect was not significant (SMD =  − 0.14; 95% CI: − 0.51, 0.24; *P* = 0.482). There was no treatment effect on triglyceride levels in broilers. However, the HDL value of broilers on dietary FTLM intervention was numerically higher than broilers on the control. Likewise, LDL value in broilers fed FTLM was numerically lower than those on the control diet. The effect of dietary FTLM intervention on intestinal histomorphology of broilers fed FTLM-based diets is shown in Table 6. Birds on dietary FTLM supplementation had significantly lower crypt depth (CD) and higher villi height (VH) and VH/CD of the duodenum than the control. There was treatment on the histoarchitecture of the jejunum and ileum with broilers fed FTLM having lower CD and VH/CD values than the control. In contrast, dietary FTLM supplementation had no effect on VH values of the jejunum and ileum in broilers.

**Table 5:** Blood lipid profiles of broilers on FTLM

Outcomes	Random-effects model					Heterogeneity			
	Dataset	SMD	95% CI		*P* value	τ^2^	Q	*P* val	*I*^*2*^
Cholesterol	14	− 0.14	− 0.51	0.24	0.482	0.45	168.74	< 0.001	92
Triglycerides	14	0.51	− 0.51	1.52	0.331	3.60	843.51	< 0.001	98
HDL	14	0.22	− 0.03	0.47	0.079	0.17	71.11	< 0.001	82
LDL	14	− 0.24	− 0.73	0.25	0.330	0.80	267.12	< 0.001	95

*SMD* Standardised mean difference; *FTLM* Fermented tropical leaf meal; *HDL* High-density lipoprotein; *LDL* Low-density lipoprotein; *CI* Confidence interval. *I*^*2*^ Inconsistency index; Q *χ*^*2*^ statistic; τ^2^ Heterogeneity variance of the true effect sizes *p* Probability

**Table 6:** Intestinal histomorphology of broilers on dietary FTLM supplementation

Outcomes	Random-effects model					Heterogeneity			
	Dataset	SMD	95% CI		*P* value	τ^2^	Q	*P* value	*I*^*2*^
Duodenum									
VH	11	1.09	0.84	1.33	< 0.001	0.14	49.89	< 0.001	80
CD	11	− 0.43	− 0.75	− 0.11	0.008	0.26	93.14	< 0.001	89
VH/CD ratio	11	1.16	0.90	1.42	< 0.001	0.15	52.73	< 0.001	81
Jejenum									
VH	11	0.03	− 0.76	0.81	0.950	1.73	485.52	< 0.001	98
CD	11	− 0.33	− 0.76	− 0.10	0.134	0.49	163.28	< 0.001	94
VH/CD ratio	11	1.86	1.38	2.34	< 0.001	0.60	150.91	< 0.001	93
Ileum									
VH	11	1.16	− 0.06	0.38	0.158	0.11	46.76	< 0.001	79
CD	11	− 0.66	− 0.95	− 0.37	< 0.001	0.20	72.94	< 0.001	86
VH/CD ratio	11	1.18	0.76	1.59	< 0.001	0.46	137.38	< 0.001	93

*VH* Villi height; *FTLM* Fermented tropical leaf meal; *SMD* Standardised mean difference; *CD* Crypt depth; *VH/CD* Villi height/ crypt depth ratio; *CI* Confidence interval. *I*^*2*^ Inconsistency index; Q *χ*^*2*^ statistic; τ^2^ Heterogeneity variance of the true effect sizes; *P* Probability

### Slaughter performance and meat quality

The slaughter performance of broilers fed FTLM-based diets is illustrated in Table 7. There was no significant difference in dressing percentage between broilers in the control and the treatment groups. In contrast, there was a treatment effect on thigh muscle weight (SMD = 0.50; 95% CI: 0.08, 0.92; *P* = 0.020) and breast muscle weight (SMD = 0.86; 95% CI: 0.48, 1.24; *P* < 0.001) between the groups. The abdominal fat weight was lower on the FTLM diet (SMD =  − 0.63; 95% CI: − 0.90, − 0.35; *P* < 0.001) than the control. The influence of FTLM on the breast muscle quality of broilers is shown in Table 8. The breast muscle of broilers fed FTLM had significantly higher pH and reduced drip loss, shear force, and b* compared with the control. The breast muscle of broiler chickens offered FTLM had numerically higher L* and a* values than the control. There was a significant treatment effect on pH, drip loss, and shear force, with thigh muscles from broilers fed FTLM having significantly higher pH (SMD = 1.85; 95% CI: 0.96, 2.74; *P* < 0.001), and significantly lower drip loss (SMD =  − 0.68; 95% CI: − 1.14, − 0.22; *P* = 0.004) and shear force (SMD =  − 0.22; 95% CI: − 0.39, − 0.05; *P* = 0.010) when compared with the control (Table 9). The L* and b* values of thigh muscle were reduced by dietary FTLM. In contrast, there was no effect of FTLM on the a* value of broilers.

**Table 7:** Carcass characteristics and abdominal fat content of broilers fed FTLM

Outcomes	Random-effects model					Heterogeneity			
	Dataset	SMD	95% CI		*P* value	τ^2^	Q	*P* value	*I*^*2*^
DP	18	− 0.35	− 0.82	0.12	0.143	0.97	453.47	< 0.001	96
Breast	11	0.86	0.48	1.24	< 0.001	0.38	139.97	< 0.001	93
Thigh	11	0.50	0.08	0.92	0.020	0.48	181.39	< 0.001	94
Abdominal fat	14	− 0.63	− 0.90	− 0.35	< 0.001	0.25	125.06	< 0.001	90

*SMD* Standardised mean difference; *FTLM* Fermented tropical leaf meal; *DP* Dressing percentage; *CI* Confidence interval. *I*^*2*^ Inconsistency index; Q *χ*^*2*^ statistic; τ^2^ Heterogeneity variance of the true effect sizes; *P* Probability

**Table 8:** Breast muscle quality of broilers on dietary FTLM supplementation

Outcomes	Random-effects model					Heterogeneity			
	Dataset	SMD	95% CI		*P* value	τ^2^	Q	*P* value	*I*^*2*^
pH	14	0.75	0.18	1.33	0.010	1.16	499.55	< 0.001	97
Drip loss	14	− 1.58	− 2.04	− 1.11	< 0.001	0.74	293.35	< 0.001	96
Shear force	14	− 0.56	− 0.85	− 0.27	< 0.001	0.27	138.07	< 0.001	91
L*	14	0.33	− 0.18	0.83	0.210	0.91	407.89	< 0.001	97
a*	14	0.03	− 0.47	0.53	0.909	0.87	392.30	< 0.001	97
b*	14	− 0.31	− 0.57	− 0.06	0.017	0.21	111.17	< 0.001	88

*SMD* Standardised mean difference; *FTLM* Fermented tropical leaf meal; L* Lightness; a* Redness; b* Yellowness; *DP* Dressing percentage; *CI*: Confidence interval. *I*^*2*^: Inconsistency index; Q *χ*^*2*^ statistic; τ^2^ Heterogeneity variance of the true effect sizes; *p* Probability

**Table 9:** Thigh muscle quality of broilers fed FTLM

Outcomes	Random-effects model					Heterogeneity			
	Dataset	SMD	95% CI		*P* value	τ^2^	Q	*P* value	*I*^*2*^
pH	14	1.85	0.96	2.74	< 0.001	2.21	677.06	< 0.001	99
Drip loss	14	− 0.68	− 1.14	− 0.22	0.004	0.57	215.03	< 0.001	95
Shear force	14	− 0.22	− 0.39	− 0.05	0.010	0.05	30.44	< 0.001	67
L*	14	− 0.45	− 0.83	− 0.06	0.023	0.39	153.95	< 0.001	94
a*	14	− 0.09	− 0.47	0.29	0.630	0.38	152.05	< 0.001	93
b*	14	− 1.49	− 2.38	− 0.61	< 0.001	2.18	675.92	< 0.001	99

*SMD* Standardised mean difference; *FTLM* Fermented tropical leaf meal; L* Lightness; a* Redness; b* Yellowness; *DP* Dressing percentage; *CI* Confidence interval. *I*^*2*^: Inconsistency index; Q *χ*^*2*^ statistic; τ^2^ Heterogeneity variance of the true effect sizes; *P* Probability

### Moderator and publication bias analyses

Table 10 shows that supplementation levels is not a significant predictor of FI (*P* = 0.427; R^2^ = 0%), FCR (*P* = 0.076; R^2^ = 4%), and ADG (*P* = 0.249; R^2^ = 3%) in broilers. Likewise, rearing phase is not a significant predictor of FI (*P* = 0.124; R^2^ = 3%) and ADG (*P* = 0.096; R^2^ = 3%) in broilers. In contrast, there was a significant relationship between FI and aspects of analysed moderators. Results indicate that broiler strains (*P* = 0.001; R^2^ = 70%), LMT (*P* = 0.001; R^2^ = 72%), and fermentation microbes (*P* = 0.001; R^2^ = 74%) were significant predictors of ADG in broilers fed FTLM. Table 11 shows evidence of publication bias as the observed significance was lower than the target significance of 0.05 for FI, FCR, and ADG.

**Table 10:** Meta-regression of growth performance of broilers fed FTLM

Outcomes	Moderators	*k*	SMD	Q	*P* value	*I*^*2*^ (%)	QM	R^2^ (%)
Feed intake	Broiler strains	84	0.09	16.0	0.003	81	16.0	16
	SL	81	0.12	1.70	0.427	84	1.70	0
	LMT	84	0.09	15.8	0.001	82	15.8	15
	Rearing phase	84	0.23	4.17	0.124	83	4.17	3
	Microbes	84	− 0.06	27.0	0.003	80	27.3	23
FCR	Broiler strains	83	− 0.26	7.10	0.029	86	7.10	6
	SL	80	− 0.26	5.16	0.076	86	5.16	4
	LMT	83	− 0.24	8.18	0.043	86	8.18	6
	Rearing phase	83	− 0.31	6.83	0.033	85	6.83	6
	Microbes	83	-0.35	16.0	0.025	85	16.0	11
ADG	Broiler strains	81	0.29	125.0	0.001	69	124.7	70
	SL	78	0.33	2.78	0.249	88	2.78	3
	LMT	81	0.27	132.0	0.001	68	132.2	72
	Rearing phase	81	0.41	4.69	0.096	88	4.69	3
	Microbes	81	0.24	148.0	0.001	66	147.6	74

*FTLM* Fermented tropical leaf meal; *SL* Supplementation level; *ADG* Average daily gain; *FCR* Feed conversion ratio; tau^2^ Estimated amount of residual heterogeneity; *k* Study number; *I*^*2*^ Residual heterogeneity / unaccounted variability; *SMD* Standardised mean difference; R^2^ Amount of heterogeneity accounted for; *QM* Moderators coefficient; *P* Probability

**Table 11:** Analysis of publication bias

Outcomes	k	SMD	OS	TS	Nfs	Number of comparison (*n*)	5*n + 10
Feed intake	84	0.1489	< 0.0001	0.05	1299	84	430
Feed conversion ratio	83	-0.1883	< 0.0001	0.05	2063	83	425
Average daily gain	84	0.3291	< 0.0001	0.05	6090	81	415

*SMD* Standardised mean difference; *OS* Observed significance; *TS* Target significance; *n* Number of study; *Nfs* Fail-safe number. *k* Study number

## Discussion

The use of TLMs as feed resources in poultry production is on the increase due to their health and nutritional benefits (Sugiharto 2021; Shi et al. 2021). However, their large-scale use in broiler production is limited due to the fact the matured leaves are high in crude fibre and moderate in ANFs such as trypsin inhibitors, pectin, phytate, and tannins (Pachauri et al. 2017). Fermentation, particularly microbial fermentation, has received attention in recent times, due to its ability to reduce dietary crude fibre and anti-nutritional factors, increase nutritional and functional properties of leaf meals, and improve the growth performance of broilers (Missotten et al. 2013; Chukwukaelo et al. 2018; Yan et al. 2019). Tropical leaves are also rich sources of polysaccharides, unidentified growth factors, and flavonoids (Ndubuaku et al. 2014; Modisaojang-Mojanaja et al. 2019). Improved digestion and uptake of nutrients from the gastrointestinal tract of chickens may play a significant part in enhancing growth performance in chickens. The observed improvement in growth performance in broilers fed FTLM may be linked to a decrease in ANF levels in the diet, as well as the breakdown of complex biomass into small units by fermentation microorganisms, which is readily utilised by the birds (Shi et al. 2021; Sugiharto 2021). This finding is consistent with previous research that found superior FCR and increased ADG in chickens fed fermented herbal products (Shi et al. 2021; Sugiharto 2021). The increased ADG and FCR in broilers fed FTLM may be related to an improvement in intestinal health and function. The intestine is the principal site for immunity, nutrient digestion, and uptake in animals (Ogbuewu and Mbajiorgu 2022). The micro-anatomy of the intestine can give insight into intestinal health. Villus height, CD, and VH/CD value are the key parameters for assessing intestinal health and functions in poultry and livestock (Niu et al. 2019, 2022). To our knowledge, higher VH and VH/CD values and lower CD indicate a better ability of the intestine to absorb and utilise nutrients (Ogbuewu and Mbajiorgu 2022). The results of the present meta-analysis suggest that FTLM increased duodenum VH, duodenum, jejunum, and ileum VH/CD values and reduced duodenum, jejunum, and ileum CD values in broilers fed FTLM. These findings were in harmony with previous reports of others (Sugiharto 2021; Niu et al. 2022), which demonstrated improved absorptive capacity and functions of the small intestine of poultry offered fermented herbal products. In the current study, the increased growth performance of broilers offered FTLM is consistent with increased VH and VH/CD values and decreased CD. This implies that FTLM boosts broiler growth performance by increasing the absorptive capacity of the small intestine.

Blood biochemical indices reflect the function and metabolism of ingested feed in the animal body (Hu et al. 2015; Ogbuewu et al. 2015). There is a positive relationship between dietary intake and blood lipids in chickens (Ogbuewu et al. 2015). Low-density lipoprotein and HDL are the two major transport proteins for cholesterol in plasma. Earlier research has shown that flavonoids in fermented leaves can regulate lipid metabolism and reduce lipid accumulation (Wei et al. 2014), and this may explain the non-significant effect of FTLM on blood lipids of broilers. These results are consistent with the findings of Santoso et al. (2015), who found that fermented *S. androgynus* leaves did not affect blood lipids in broilers. In converse, Kamalia et al. (2014) stated that inclusion of unfermented herbal products in the chicken diet reduced the concentrations of blood cholesterol, triglyceride, and LDL, but elevated HDL content in broilers. The observed variation could be attributed to the fact that TLM used in the present meta-analysis was fermented.

Slaughter performance indicates the ability of animals to convert feed to muscle tissues. Muscle and visceral (abdominal) fat are the key indices to measure carcass yield and meat quality in broilers. In the current meta-analysis, the addition of FTLM to the broiler diets increased the weights of breast and thigh muscles. This finding is consistent with the results of Yang et al. (2008), who observed an increased in cut part (breast and thigh) weights of broilers fed fermented herbal products. This increase in cut-part weights was most likely due to the quality of FTLM-based diets leading to improvements in muscle protein accretion. Increased visceral fat deposition in broilers has a direct impact on processed meat products, lowering carcass yield, meat quality, and consumers’ purchase desire, as well as reducing economic benefits (Yu et al. 2019). Furthermore, increased abdominal fat deposition in broilers indicates poor dietary energy use efficiency. This study indicates that FTLM reduced abdominal fat yield in broilers, indicating the ability of bioactive compounds present in FTLM, especially flavonoids to regulate fat metabolism in broilers. Similarly, Ding et al. (2021) found reduced abdominal fat yield in broilers fed fermented herbal products. This finding suggests that the presence of probiotic organisms and functional bioactive compounds in FTLM might have influenced the lipid metabolic pathway, preventing fat deposition in the abdomen (Sugiharto 2021).

Muscle pH is one of the parameters affecting muscle characteristics post-slaughtering (Aberle et al. 2001). Furthermore, a rapid reduction in postmortem pH can result in protein denaturation that may lead to pale colour and poor water holding capacity (Cao et al. 2012). In the present study, FTLM improved the breast and thigh muscle pH values of broilers. This implies that FTLM can maintain the pH value of breast and thigh muscles in broilers. The pH obtained in this study was within the normal values of 5.70 and 5.96 reported by Fletcher et al. (2000) in broiler meat. Drip loss and cooking loss are markers used to determine the ability of muscles to retain water, i.e. water holding capacity (WHC). The significantly reduced drip loss in breast and thigh muscles of broilers offered FTLM compared to the controls, suggests an improvement in WHC of the meat. The improved drip loss suggests a reduction in the nutritional value via exudates that were released, and this resulted in firm and tender meat (Dabes 2001). Shear force (tenderness) is one of the important organoleptic parameters that determine consumer acceptability (Miller et al. 2001; Kannan et al. 2002). In this study, the results revealed that drip loss and shear force are consistent with that of pH values. The improvement in shear force may be related to enhanced antioxidative status, and flavonoids may play a beneficial role. Meat colour is a measure of quality and is vital due to its link with consumer acceptability (Pelicano et al. 2003). This study revealed that FTLM had similar breast muscle L* and a* values, but reduced the b* value. Broiler meat is also rich in polyunsaturated fatty acids making them susceptible to free radical attack (Arshad et al. 2011). Thus, the antioxidant status of muscles affects the meat quality. Dietary FTLM significantly reduced L*, a*, and b* values of thigh muscle. The likely cause of the significantly lower L ∗ value in broiler thigh muscle might be related to the increased antioxidant activity of FTLM, which protects muscle cells from oxidative damage and inhibits cell sap extravasation.

### Moderator analysis and publication bias

This study revealed that broiler strains, LMT, and fermentation microbes were significant predictors of feed intake and accounted for about 16, 15, and 23% of variations in feed intake. Results also suggest that the Arbor Acres strain had a higher ability to utilise FTLM diets than the Lohmann strain, showing that Arbor Acres strain fed FTLM-supplemented diets was superior to the Lohmann strain in terms of feed intake, FCR, and ADG. These findings support the view that the genetics of chicken influence its production parameters (Sebola et al. 2015). Results show a small effect of LMT for feed intake, FCR and a large effect for ADG. This suggests that LMT is a significant predictor of ADG in broilers and accounted for most of the sources of heterogeneity. The significantly higher feed intake in broilers fed fermented *G. biloba* leaf meal compared with those fed fermented *S. androgynus* leaf meal, indicates the quality of the fermented *G. biloba* leaf meal.

The improved growth performance of broilers fed fermented TLM at 0 – 5 g/kg feed compared to control agrees with the earlier reports of Ding et al. (2021), who found sub-optimal performance of broilers offered high doses of FTLM due to the dilution of basic nutrients in the diet. This finding is consistent with the reports of Niu et al. (2019) who reported decreased FI and ADG in broilers fed high doses of fermented *G. biloba* leaf meal. Results also demonstrated that fermentation microbes is a limiting factor in this study and can lead to variable results in FI, FCR, and ADG among trials included in the meta-analysis. The significantly higher feed intake recorded in broilers fed TLM fermented with a mixture of A*. niger* and *C. utilis* when compared to those fed TLM individually fermented A*. niger* and *T. harzianum* indicate the high ability of these microbes to improve the quality of TLM during fermentation. There is little effect for rearing phase as a moderator for FCR. The percentage of heterogeneity not accounted for by the analysed moderators could be to the following factors: seasonal variations, storage conditions of the leaves, and stocking density not analysed in the meta-analysis. There is evidence of publication bias for FI, ADG, and FCR. The Rosenberg Nfs for the database was about 3, 5, and 15 folds higher than the thresholds needed to consider the mean effect size for FI, FCR, and ADG valid. As a result, publication bias was not an issue in this study because a large number of unpublished studies would be needed to alter the statistically significant effects of FTLM on FI, FCR, and ADG in broilers.

## Conclusion

The results of this investigation demonstrated the potential of dietary FTLM supplementation to improve growth performance, intestinal histomorphology, slaughter performance, and meat quality of broilers without negative consequences on dressing percentage and blood lipid profiles. Restricted subgroup analysis results indicate that supplementation of FTLM at 0—5 g/kg feed is associated with significant improvement in growth performance parameters of broilers. However, future research should focus on determining the actual supplementation levels of FTLM that optimised all the production parameters of broilers. Meta-regression showed that analysed moderator variables were significant predictors of treatment effect and accounted for some of the sources of heterogeneity. The findings of the meta-analysis will help poultry farmers, animal nutritionists, and policy-makers to make an informed decision on the potential of FTLM to improve broiler productivity and meat quality.

## Data Availability

Data will be made available on reasonable request.
